# Using the NIATx Model to Implement User-Centered Design of Technology for Older Adults

**DOI:** 10.2196/humanfactors.4853

**Published:** 2016-01-14

**Authors:** David H Gustafson Jr, Adam Maus, Julianne Judkins, Susan Dinauer, Andrew Isham, Roberta Johnson, Gina Landucci, Amy K Atwood

**Affiliations:** ^1^ Center for Health Enhancement Systems Studies Department of Industrial and Systems Engineering University of Wisconsin - Madison Madison, WI United States

**Keywords:** eHealth, user-centered design, technology, aging in place, independent living, consumer participation, accessibility

## Abstract

What models can effectively guide the creation of eHealth and mHealth technologies? This paper describes the use of the NIATx model as a framework for the user-centered design of a new technology for older adults. The NIATx model is a simple framework of process improvement based on the following principles derived from an analysis of decades of research from various industries about why some projects fail and others succeed: (1) Understand and involve the customer; (2) fix key problems; (3) pick an influential change leader; (4) get ideas from outside the field; (5) use rapid-cycle testing. This paper describes the use of these principles in technology development, the strengths and challenges of using this approach in this context, and lessons learned from the process. Overall, the NIATx model enabled us to produce a user-focused technology that the anecdotal evidence available so far suggests is engaging and useful to older adults. The first and fourth principles were especially important in developing the technology; the fourth proved the most challenging to use.

## Introduction

In 2010, the Federal Agency for Healthcare Research and Quality started funding the Active Aging Research Center (AARC) to develop technology to help older adults live longer independently [1]. The AARC is housed at the Center for Health Enhancement Systems Studies (CHESS) at the University of Wisconsin-Madison. CHESS has been building and testing information and communication technologies (ICTs) for patients and their families since the 1970s. Previous CHESS ICTs have been proven effective in numerous randomized trials for a variety of conditions, including alcohol use disorders [2], lung cancer [3,4], pediatric asthma [5], breast cancer [6], and HIV [7].

In the original AARC grant application, we defined what the technology for older adults would accomplish, building on previous CHESS systems, but did not identify a specific technological solution. Instead, we planned to develop a technology for adults aged 65 and over by working closely with older adults themselves as well as with informal caregivers, health care professionals, community members, and others; test the technology in a randomized controlled trial [8]; and, if the technology proved to be effective, disseminate it.

One assumption we had when we started this work was that older adults are rarely the target of technology development. Although guidelines exist for designing technology for older adults [9] and the literature reports some efforts to develop technology with and for older adults [10,11], we found few easy-to-use websites and interfaces designed specifically for older adults. Yet recent research shows a significant increase in technology use among older adults. Between 2008 and 2012, adults aged over 65 had an increase of 39% in Internet use, the largest among all age groups, and now 50% of all older adults are online [12]. Older adults are willing to use technology if they think it adds value and convenience to their lives and supports their activities [13]. While older adults have physical limitations that can make using technology challenging, such as low vision, dexterity problems, and cognitive issues [14], we believed that designing a technology with and for older adults would help overcome barriers to use [15].

This paper reports on using a customer-focused process improvement model (Network for the Improvement of Addiction Treatment [NIATx]) as the user-centered design (UCD) approach to developing technology for older adults.

## UCD at CHESS

Central to UCD is the principle that having a thorough understanding of the end user’s needs and capabilities is essential to creating the most effective system or product [16]. Since the late 1980s, when early publications [17] sparked an interest in applying UCD to technology development, various methods and models of UCD have been extensively researched [18]. Despite the work done on UCD research and application, UCD remains loosely and variously defined [18].

At CHESS, the tech team used UCD methods (usability testing, card sorting, paper prototyping, focus groups, surveys, etc [19]) without having a structure for using these methods at different stages of development. In addition, we often ran out of time or lacked the resources to implement UCD methods throughout a project. As UCD expert Jakob Nielsen [20] pointed out, many developers abandon UCD methods because of cost, time, and complexity, and this was the case at CHESS. Although we agree with Karat [21] that UCD does not need to be a rigid set of practices, we sought practical key principles that would provide a structure for the application of UCD methods throughout the life cycle of product development. Gulliksen et al [22] defined 12 key principles of UCD based on standards and experience in using various models in a variety of projects. Their work also includes lists of activities that relate to each principle. Even with these well thought out and researched principles, we became overwhelmed with the options and activities that could be used and lacked the time and resources to research alternative models of UCD.

The tech team at CHESS is relatively small, with 2 software developers; 1 user-interface designer; 1 Web master; 2 information technology professionals supporting hardware, infrastructure, and the helpline; and the tech director. Each tech project usually involves a manager, a software developer, and a user-interface designer. In addition, tech team members are a shared resource at CHESS, meaning that individuals usually work on multiple projects at one time. Because of the size of the AARC grant and the number of different goals, academic departments, and principal investigators (PIs) involved, we felt we needed clear guidelines on how to apply UCD within the development process, so that we could incorporate user feedback in design decisions, promote speed, and keep team members informed about progress. Table 1 shows the organization of the AARC project. The technology developed during the project ultimately became a website called “Elder Tree.”

**Table 1:** Organization of the Active Aging Research Center project.

Center individual or subgroup	Main functions
Lead principal investigator (PI)	Generates ideas, overall management and priorities, final decision making. The lead PI is the Director of Center for Health Enhancement Systems Studies, where the Active Aging Research Center is housed.
Project director	Day-to-day management of overall project
Research teams	Each team works on 1 of the following 5 challenges for older adults: isolation and loneliness, driving and transportation, caregiving, medication management, and falls prevention. Each team has a PI, change leader, and team members.
Community partners	Identify the needs and assets of older adults; provide feedback on evolving iterations of the Elder Tree technology. Community partners are older adults, the Wisconsin Institute for Health Aging, and local Aging and Disability Resource Centers.
National Advisory Committee	Review of plans and progress, advice on Elder Tree technology and research. The committee consists of 17 nationally recognized advisers in gerontology, technology, public policy, medicine, communications, driving and highway safety, organizational change, and other areas.
Tech team	Design and development of information and communication technologies for patients and family members, including Elder Tree.
County coordinators	Local management of the randomized trial (recruitment, training, etc). County coordinators are grant-funded employees, 1 in each of the 3 regions where Elder Tree is being tested.
Strategy teams	Through interviews, identify needs and assets of older adults in each community. Strategy teams consisted of citizens from each of the 3 regions where Elder Tree is being tested.

## The NIATx Model

The NIATx model, developed at CHESS, is a simple framework for process improvement. NIATx originally stood for “Network for the Improvement of Addiction Treatment”; the NIATx model was initially used to improve retention and access to care in behavioral health agencies [23]. Now NIATx functions as a word referring to a division of CHESS that teaches and conducts process improvement in a range of health care and other organizations.

The model, which has only 5 principles, was intended to be easy to learn and implement so that individuals with little or no knowledge of process improvement can quickly test changes to improve services and outcomes. The model has no levels of training or complicated data elements to collect. Although not specific to technology development, the model is a user-centered approach intended to be applied flexibly [23,24]. The NIATx model is evidence based, and many of us on the tech team were familiar with it from developing tools to support NIATx research projects conducted at CHESS. Unlike other models of UCD, the NIATx model includes a method for developing innovative solutions. For these reasons—ease of use, flexibility, familiarity with the model, the model’s evidence base, and its approach for developing innovative solutions—we decided to apply the NIATx model as a UCD framework that would give structure to the development process.

### The Five Principles of the NIATx Model

The NIATx model rests on 5 principles that have been shown to be the essential elements of successful change projects [25]. These principles were developed from analyzing decades of research from 13 different industries related to why some projects fail and others succeed [25].

As we considered using the NIATx model as a framework for UCD, 3 of the principles—rapid-cycle testing, understanding and involving the customer (or end user), and getting ideas from outside the field—seemed especially useful because they would enable us to test innovative ideas quickly, assess their effectiveness, incorporate user feedback, and make additional changes rapidly.

The first principle, *understand and involve the customer,* is the most important principle of the NIATx model. In fact, this principle has more impact than all the other principles combined [25]. “Customer” refers to the end user, who may or may not pay for the product or service being designed or implemented. Allocating the time and resources to deeply understand the needs and assets of end users and getting regular ongoing end user feedback increase the likelihood that a product will succeed. In this paper, we refer to the customer as the “end user” or “older adult.”

The second principle, *fix key problems,* arises from the understanding that a project is more likely to succeed if top management is involved and committed to the project, in part because this makes it more likely that the support and resources needed to succeed will be available. One way to ensure the commitment of top management is to address the key issues or problems that top managers face. One strategy is to ask, “What keeps the CEO awake at night?” The answer identifies the problem(s) to start with.

The third principle is to *pick an influential change leader*. The role of the change leader is to move a project forward by identifying and removing barriers to progress. An influential change leader is a staff member who has respect from management and staff, is a good leader, and has direct access to the CEO and other critical stakeholders. The change leader should have the authority to do whatever it takes to keep a project moving forward.

The fourth principle, *get ideas from outside the field,* is the second most important NIATx principle [25]. It can be broken down into 3 phases. First, identify a field or fields that face problems similar to the problems your organization faces. Second, find the organization in that field that is best at dealing with that problem. Third, identify what makes that organization so much better than others at addressing that problem. This process forces you to identify the core problem you are facing and can lead to innovative solutions. Atul Gawande [26] used this principle when he described the possible application of coaching, as done in sports and music, to the work of surgeons and the application of cost and quality control, as done in restaurant chains, to health care [27]. Looking outside the field of UCD for an approach to technological development, as we did in this case, can be considered another example of this principle.

As an example of using this principle, staff members who wanted to improve teamwork in their organization would begin by asking, “What industry requires good teamwork?” One answer might be National Association for Stock Car Auto Racing (NASCAR); the pit crew of a NASCAR team demonstrates exceptionally good teamwork. One particularly good pit crew works for NASCAR driver Denny Hamlin [28]. Staff members then identify the key characteristics of Hamlin’s crew that make the crew successful. For instance, crew members work together seamlessly under very stressful conditions. Each member has a clearly defined job, and each understands every other member’s job. Pit crews constantly practice to stay sharp for the race, and their performance is constantly measured. With these characteristics identified, staff members can now apply the ideas to their own work environment. Looking outside the field can produce solutions not previously considered.

The fifth principle, *use rapid-cycle testing,* encourages developers to develop small improvements and test them with end users and stakeholders to see how they work. After each test, the improvement (or in our case, tech) team makes changes and then tests again. Several cycles of rapid-cycle testing help create a high-quality product on release or an improvement in a process that actually works. Rapid-cycle testing was first described by Shewhart [29] and revised and popularized by Deming [30]. For example, if stakeholders identified reducing the home page bounce rate (the rate at which users abandon a website after landing on the first page) as a key problem to solve, usability tests would be used early in development to determine what seems to cause users to leave the home page. The answer(s) would determine changes to make in the home page, and the success of the changes would be determined by another wave of usability testing. The process would continue until stakeholders were satisfied that the bounce rate had been sufficiently reduced.

## Applying the NIATx Model

### Understand and Involve the Customer

Before starting to develop a technology to help older adults continue to live independently, we began the process of understanding our end user. What we learned drove development, including what services to provide in the technology, what content to include, and the overall design of the system [31]. The Elder Tree website in use in the randomized trial as of this writing is available in an archived version [32].

All staff members working on the AARC project (PIs, researchers, tech team members, administrative staff—see Table 1) were asked to take part in focus groups or one-on-one interviews with older adults and their caregivers. During the course of the project, hundreds of interviews and more than 20 focus groups were conducted. All staff were asked to write, for each individual they talked to, a story that summarized the older adult’s experiences and current situation. Creating stories from these interviews brought the experiences of older adults to life, giving us a better understanding of and greater empathy with their day-to-day challenges and joys. The AARC staff members met regularly to share and discuss the stories. From defining high-level goals to conceptualizing and developing solutions, these stories were foundational to our development work.

During this period when all project staff members were getting to know firsthand the assets and needs of older adults, tech team members volunteered at a local senior center to better understand how older adults use and learn to use new technology. Members of the tech team designed and taught a series of classes on using computers and the Internet. Topics included Internet basics, Internet safety, Facebook, Skype, and downloading and managing digital images. While a tech team member led a class, other team members circulated among the students to offer one-on-one support. Seeing and experiencing, up close, how older adults interact with technology had a profound effect on our work. For example, we observed that arthritic hands had trouble using a mouse and that Web pages with many sections and subsections were hard for some older adults to understand and hard for others to see. These and many similar observations directly influenced the design of the Elder Tree website.

From all of these interactions with older adults, a few issues surfaced repeatedly. Older adults frequently expressed a concern about their safety on the Internet. They did not want to get scammed, lose money, or be asked to give private information. It also became clear we would need to address older adults’ decreasing motor dexterity and problems with vision and hearing both in the computer we selected for older adults to use and the interface design. During the development of the technology, we often found ourselves rejecting accepted Internet conventions for the sake of accessibility. For example, we decided not to use rollover effects to display additional information. Users who struggle with a mouse can be distracted and disoriented by the rollover effects as they navigate a page. We also rejected a dashboard-type home page that would give a dense display of information. Instead, we embraced a simplified design with the goal of having a single task per page.

The process of understanding the end user produced another effect: It forged personal connections between tech team members and older adults and turned all of us tech team members into advocates for our end users.

### Fix Key Problems

One way to identify key problems is to ask, “What keeps the CEO awake at night?” These problems are good ones to start with because top management will more likely be engaged in a project that addresses these problems and give the project the attention and resources it needs to be successful. Applying this principle to developing technology in our grant-funded project required some modification. The overall goal of the grant was to help older adults live longer independently. The PIs who applied for the grant took this as their mission; we regarded it as the answer to what was keeping the CEO up at night. However, this statement of the problem was too broad to suggest specific development steps, so we asked older adults themselves to help identify key problems more specifically.

In addition to the work done at the beginning of the project described earlier (conducting focus groups and interviews with older adults and summarizing the results in stories, and volunteering to teach older adults about the use of technology at a senior center), early work on the AARC project included a process called Asset-Based Community Development (ABCD) to learn about older adults in their communities and lay the foundation for dissemination [33]. Because our grant-funded project would culminate in testing whatever technology we developed in a randomized trial in 3 regions of Wisconsin (urban, suburban, and rural), we implemented ABCD in 1 community in each of the 3 regions. A staff member from CHESS with extensive experience in using ABCD worked with the county coordinator—someone who lives in the target area and was hired with grant funds to manage the project locally—to implement ABCD. The CHESS researcher and county coordinators led the creation of strategy teams made up of citizens from each community. These citizens came from local agencies, businesses, institutions, and organizations. Strategy team members and other volunteers interviewed friends and neighbors to create an inventory of the assets, challenges, and aspirations of older adults and their caregivers in the community. In all 3 regions, 80 home visits were conducted; many more focus groups and interviews took place in other settings. Remarkably, the top 3 problems for older adults were the same in each of the 3 communities: isolation and loneliness, not knowing about community resources and events, and transportation to and from resources and events.

Throughout the development work, we also relied on the expertise of researchers and community partners to inform our work (Table 1). Researchers came from the fields of falls prevention, geriatrics, driving, transportation, and innovation. A National Advisory Committee consisted of leading thinkers from these and other fields. This committee met annually to advise us on the progress and direction of the project. Community partners, researchers, and National Advisory Committee members identified other key problems affecting the ability of older adults to live independently, especially medication adherence, dementia, and depression.

Given all the problems identified by various stakeholders involved in the project, whose view of the key problems should take precedence? Were the various PIs, collectively, the CEO? Or were the community partners? What about potential payers for the final product of the study? If the technology does not address the problems of payers, they will not be willing to fund implementing the technology that results from this research. And what about older adults themselves? If we did not address their key problems, they would not use the system. All these groups had important ideas and insights that we needed to consider.

After looking at all this information, our lead PI decided that we should first develop and test something to address isolation and loneliness. This would allow us to address the top issue identified by older adults themselves; we tech team members also thought we could develop something quickly and rapidly test and retest it. AARC researchers would continue to conceptualize solutions for the other issues, such as falls prevention and medication management, but initially the tech team would focus on this particular issue.

### Get Ideas From Outside the Field

Early in our development work, we focused on reducing the number of older adults being affected by scams because older adults so often raised this issue. We looked into examples of excellence in sales, lie detection, psychic cold reading, and cognitive interviewing, collecting ideas that might be repurposed. Cold reading, which is how a psychic creates the illusion of knowing someone he or she does not know, seemed to have the greatest potential to reduce scams. Cold reading uses several techniques (eg, fishing, vagueness, push statements, switches); for each, a block can be used. We began to discuss with older adults their use of the equivalent of blocks when they used technology, but abandoned this because older adults were so pervasively concerned about scams that we instead created a closed system that could be used only by vetted participants.

We also looked for ideas outside the field when we brainstormed topics to include in the discussion group. We asked ourselves to think of other ways older adults obtained information. One of our team members suggested newspapers. We decided to base our initial topics in the discussion group on the different sections of a newspaper (eg, sports, local news, arts, entertainment). Our premise was that this organizational system would feel familiar to most older adults.

### Pick an Influential Change Leader

At CHESS, the change leader for a research project is responsible for driving a project forward and removing barriers to progress. Chosen by the CHESS director and co-director, the change leader understands and represents the needs of the end user, is committed to seeing the project succeed, has a strong belief in the value of the project, can defend his or her position articulately, and is someone other people can easily follow or find charismatic; the change leader has direct access to the PI of the study. A change leader is responsible for leading and motivating the team, organizing meetings, removing roadblocks, and delegating. Because designing and developing a new technology for older adults was a large effort involving research teams (1 team each for isolation and loneliness, driving and transportation, caregiving, and so on), a project director led the work of the research center overall, and a change leader emerged from the tech team to lead development work on each research team. Change leaders, working under the guidance of the project director, understood and represented the needs of the end user; acted as a liaison between the tech team and older adults, researchers, and other stakeholders; and removed barriers in the tech team’s way.

### Use Rapid-Cycle Testing

Quickness is the essence of rapid-cycle testing. Each test should take from a few hours up to a few weeks, depending on what is being tested [23]. Being clear about 2 things speeds testing: What are you trying to accomplish? How will you know whether the change is an improvement? Instead of taking months to design an entire system, a development team can create a piece of the system, quickly test it with users to get feedback, make changes, and retest. We used 2 methods to conduct rapid-cycle tests: (1) one-on-one usability testing and (2) pilot tests with older adults in the field. Usability testing helped us understand whether older adults could navigate the interface and allowed us to see what they did as they used it. We usually tested usability in 1 sitting with an older adult or adults, using paper prototypes or early builds of the system. Pilot tests took 2 weeks or more and involved older adults using various iterations of the system in their homes. Pilot tests allowed us to see whether users thought the system was helping them and were likely to keep using it.

Our initial development work focused on creating a simple online discussion group. We wanted to test whether older adults with limited or no experience with technology would be able to use the system. Once we had a working website, we conducted a single session of one-on-one usability tests at a local senior center. This session took just a few hours, with 3 participants taking part. From these tests we were able to identify glaring usability issues, such as how we were indicating clickable buttons.

After making changes, 10 older adult volunteers were recruited to take part in a 2-week pilot test. Participants were given a computer and access to the Internet through a mobile hotspot if they did not have their own Internet access. Participants were able to select from a number of devices, including 7-in. Android tablets, iPads, laptops, and 23-in. touchscreen all-in-one desktops. The AARC staff visited each participant at the participant’s home; set up the computer and Internet connection, if necessary; and trained each participant on how to use Elder Tree. In all, we provided a computer and Internet connection to 9 of the 10 participants. During the 2-week pilot, AARC staff acted as discussion group “seeds,” actively engaging with participants online to make sure Elder Tree had new messages and comments every day. After the 2-week pilot, each older adult was interviewed about his or her experience. This information, along with use and observational data from the pilot, was analyzed to make decisions about changes to make in the system. Work began on making those changes to run a second pilot test.

In total, 5 pilot tests were conducted involving more than 100 older adults. Eventually, we had enough older adults using the system that we were able to turn our discussion group into a beta version of the final website. Instead of running a pilot, getting feedback, making changes, and running another pilot test, we kept the site active for participants to use, solicited feedback from users in the discussion group, and rolled out improvements as they were completed. Using the discussion group for feedback proved to be an effective method of gauging the perceived usefulness and appeal of the site. The pilot tests and the beta site also supplied older adult volunteers who agreed to continue using the site during the randomized trial to act as “seeds” and peer mentors to study participants.

As we improved the site and developed new features, we continued to run ad hoc usability tests at senior centers and get ideas and feedback from researchers, experts on the National Advisory Committee, and our community partners.

## Discussion

### General Findings and User Views

Using the NIATx model has allowed us to develop a technology based on the needs and capabilities of end users. Anecdotal evidence suggests that using the technology is reducing isolation and loneliness among older adult users, which was the most important challenge older adults identified in the interviews, focus groups, and surveys we conducted. The views of some Elder Tree users are presented in Textbox 1.

**Views of some Elder Tree users.**

- Since my husband died, I rarely get out of my house and Elder Tree has saved me.
- Elder Tree has given me back a sense of belonging.
- Elder Tree has connected me with people who are going through the same challenges as me. We are there for each other...I like that.

In addition, although we are still conducting the randomized control trial, early use data indicate that older adults facing barriers to technology adoption are using the website. Technology adoption generally is lower among those with less income, those with less experience using technology, and those with the least education [34]. Looking at study participants who created content on Elder Tree, meaning those who wrote or commented on discussion group messages, we found that 20.1% (27/135) created content 5 or more times a month (Table 2). Of this group, which we call super posters, 74% (20/27) did not have a computer or Internet access before the study, 19% (5/27) had a 4-year degree or above, and 89% (24/27) found dealing with finances challenging or difficult (Table 3).

**Table 2:** Categories of content creators (N=135).

Category	n (%)
Super posters (wrote >5 messages/month after training)	27 (20.0)
Medium posters (wrote ≥1 but <5 messages/month after training)	33 (24.4)
Low posters (wrote ≤1 message/month after training)	39 (28.8)
Did not post (Never wrote a message after training)	36 (26.6)

**Table 3:** Demographic characteristics of Elder Tree users.

Characteristic	Super posters (N=27) n (%)	Medium posters (N=33) n (%)	Low posters (N=39) n (%)	Did not post (N=36) n (%)
Did not have computer with Internet connection before the study.	20 (74)	15 (46)	22 (56)	16 (44)
Education (4-year degree or above)	5 (19)	8 (24)	8 (21)	11 (31)
Find dealing with finances challenging or difficult	24 (89)	22 (67)	19 (49)	17 (47)

**Figure 1:**
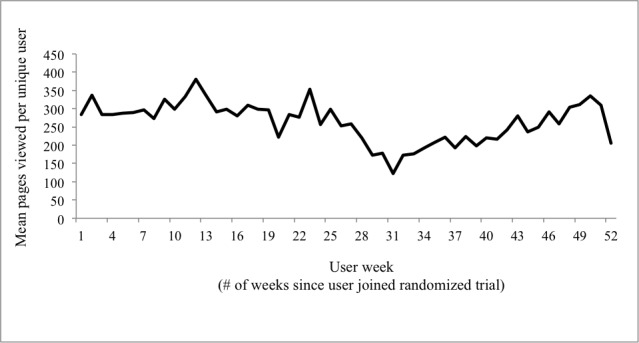
Mean Elder Tree pages viewed per user.

We also looked at the overall use of the website by all participants, not just those creating content. When looking at mean pages viewed per user (Figure 1), we see a decrease in use after 6 months on study. However, near Month 8, we see a gradual increase to levels near the start of the study.

Although the qualitative and quantitative use data suggest that our development approach has led to a technology that older adults use, we also encountered problems and learned some lessons, which are described in the following sections.

### Balance Efforts to Understand the End User Against Resources

Turning face-to-face meetings with end users into stories is a powerful exercise in understanding their needs and assets, but having everyone in a complex project (top management, tech team members, and research and administrative staff) conducting face-to-face interviews is very time consuming. We believe that the more people who see and hear from end users firsthand, the better, but we recognize that this approach might not be feasible in every organization. At the very least, 1 member of the development team, probably the change leader, needs to take the role of user advocate. This person should spend time in the field interviewing end users and conducting usability tests and be the voice of the user when conceptualizing new features and designs, therefore helping keep the project team focused on features that have the greatest efficacy.

Using a community-based process such as ABCD consumes considerable time and resources. Because we included this process in our grant application, we had the required resources for it. Although the process produced insights that helped define key problems, most organizations would not have the resources to use the process. We would rely in the future on focus groups; individual interviews with end users; creating personas to represent the different users of the technology, each with its own needs and assets; and the expertise of researchers and community partners to determine the problems faced by end users.

### Beware of the Power of An Individual’s Story

Storytelling is a powerful tool and brings the challenges of end users to life. However, individual stories can be almost too powerful. In our project, the moving story of 1 adult occasionally shut down what might otherwise have been a productive discussion of an improvement or new feature. Looking for common themes in multiple stories helps prevent a single story, or several, from having too much weight in development.

### Prioritize Ideas

Our development process produced an enormous number of proposed solutions from older adults, PIs, researchers, and community partners. We had to filter these ideas so we could spend our limited resources productively. We constantly asked ourselves, “How will this feature help an older adult continue to live independently?” Remembering the overall goal of the project served as our compass. Having a strong user advocate on staff and using rapid-cycle testing also helped us filter out nonessential improvements.

**Figure 2:**
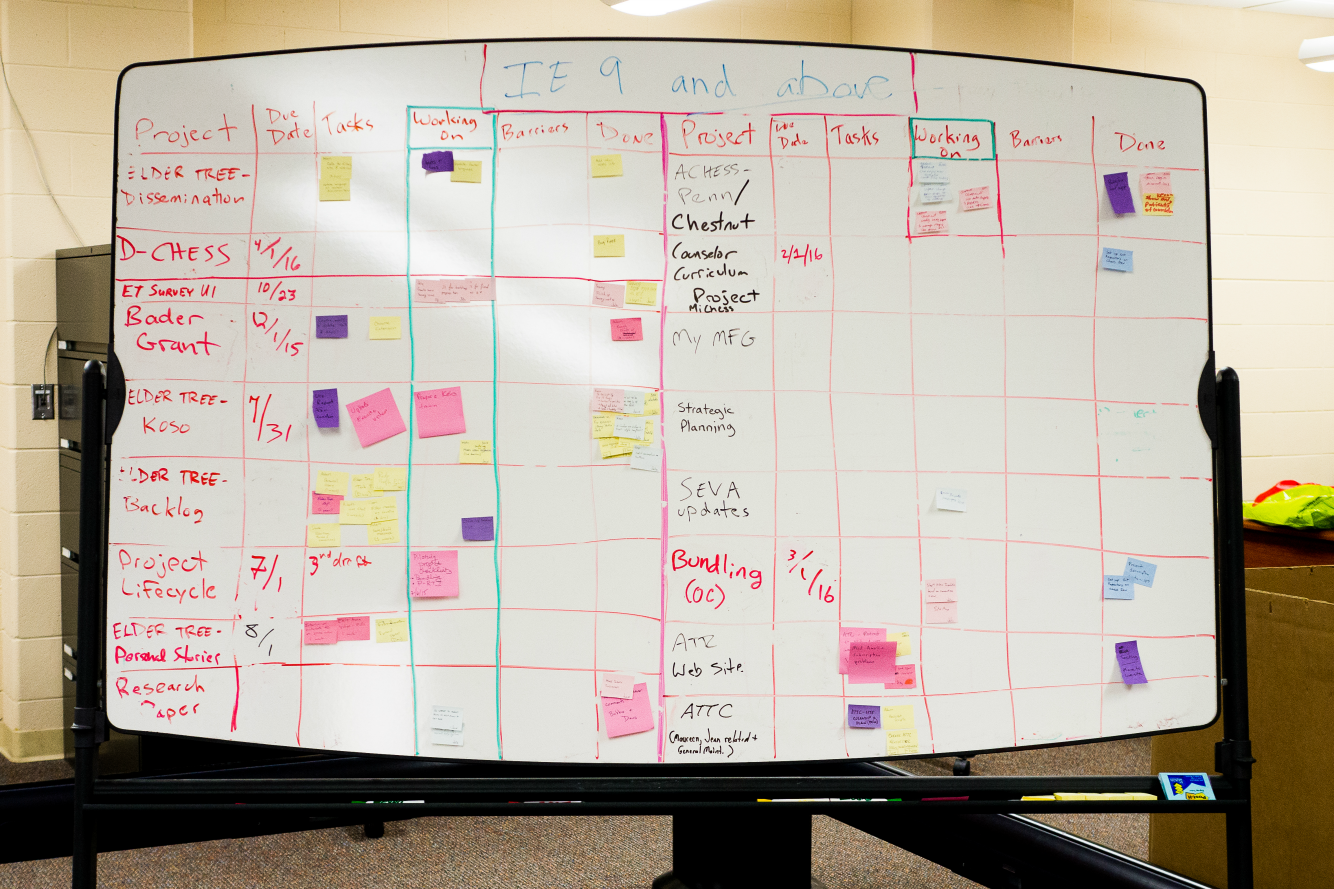
Photograph of the tech teams's project board.

To help us establish and assess priorities in the project, we used a modified agile project board (Figure 2) where we listed all the features under development with time estimates and barriers to completion. As new features were suggested, this board was an effective visual snapshot of the tech team’s workload. The board was also helpful in assessing priorities with PIs.

As this project continues, we constantly reevaluate key problems. Although older adults identified the initial key problems we addressed, other sources of information have influenced us as the project has progressed. For example, it became clear that the health care industry would need to see value in the technology if it is going to pay for sustaining it. A key problem in this industry is keeping down costs. Could the technology help detect health problems that, if identified early and treated quickly, could prevent the need for costly medications and hospitalizations? We are in the later stages of developing a reporting function for health care providers and have been told by providers and insurers that it will be a very important development.

### Get Ideas From Outside the Field

We were excited about the potential of this principle to drive innovative problem solving. However, in reality we found it difficult to implement. We used this principle occasionally, such as when we used the labels of newspaper sections as models in our work, usually when we were struggling to conceptualize a feature for Elder Tree. Using this principle in its 3 phases takes an investment of time and resources, in part because it requires reading and research. We found it impossible to use this principle spontaneously in the context of a large meeting. We do see great value in this principle for future projects and plan to continue to assess the time spent on reading and researching to apply the principle against the anticipated value of the results.

### You Can Never Have Enough Communication

Because of the organizational complexity of the project, we wanted to have a clear and effective communication plan in place. We feared that work on the individual research teams would proceed in silos. We adopted a communication plan to give the tech team direct, regular access to the PIs who led work on the research teams. The tech team supervisor also held regular meetings that included tech team members and change leaders to discuss development status, brainstorm ideas, and coordinate future development. The lead PI and project director made themselves available to attend these meetings when necessary to work through impediments to progress. Weekly and eventually biweekly steering committee meetings brought together the PIs from the research teams, the lead PI, the project director, and change leaders to update all attendees on progress, collect feedback, and discuss any barriers we were facing. Having many avenues of communication was a priority for us.

### Ensure Rapid-Cycle Testing Is Rapid and Has Clear Goals

Of the 2 types of rapid-cycle testing we conducted (ie, usability tests and pilot tests), usability tests produced more rapid results. One-on-one usability tests are comparatively inexpensive to conduct and gave us immediate feedback on usability. These tests were usually conducted in 1 day with only 1 or 2 staff members involved. We were able to evaluate results immediately and quickly make changes and test again. For this project, we used the wireframing program Lucidchart to create paper and digital prototypes to test new concepts in addition to the fully functional website.

By contrast, the 5 pilot tests we conducted evolved into large tests and produced feedback more slowly. In the future, we would be clearer about the length of each pilot test, the features of the system being tested, and the method of collecting qualitative data at the end of each test. The first pilot test we conducted took place in 1 county for 2 weeks. At the end of the 2 weeks, we visited each participant at home and conducted a survey about how it went. The next pilot test took place in 3 counties for 1 month. Again, we visited each participant and collected survey data. Each successive test had more participants. Reaching out to each participant to survey him or her on use became a scheduling and human resource challenge. Pilot tests began to take too long, and survey data were not collected in a timely manner.

While the pilot tests provided feedback on long-term, real-life use of the website and eventually led to the creation of a beta site, they were not rapid-cycle tests. Our goals for each pilot test became less clear. Instead of testing specific elements, the pilots tested the whole system, which sometimes made it difficult to pinpoint what needed to be changed. However, our experiences with usability and pilot tests led to a clearer understanding of when and where to use specific UCD methods. For example, usability tests are a good way to test individual components of a system, whereas pilot tests are good for assessing the overall value of a system. One of our colleagues has developed a model that shows the types and sequence of technology testing within a research environment. This model will help us apply UCD methods at progressive phases of development in future projects (Table 4).

**Table 4:** Isham model of technology testing sequence (from feasibility to efficacy).

Feasibility ^a^	Usability	Perceived usefulness	Efficacy
Does the concept show promise? Can it be built?	Can users navigate the interface?	Do users think the technology is helping?	Does the technology actually help users?
	Do they understand what is happening?	Do they want to keep using it?	
Test the concept using discussion, focus groups, and interviews with key stakeholders and end users.	Test navigation using paper prototypes, mock-ups, card sorting, and usability testing of early builds.	Longer pilot tests with users operating the system in their own environment.	Run a full experiment.

^a^ The stages of technology testing generally occur in the order shown in Table 4 (ie, from feasibility to efficacy). The cost of testing generally becomes more expensive from left to right.

### Conclusion

Developing the technology for this project required a constant balancing between features and simplicity. We repeatedly heard from end users that they valued simplicity over added features, while other stakeholders (community partners, PIs, National Advisory Committee members) frequently suggested adding new features. The NIATx model, with its focus on the end user, allowed us to keep the interests of older adults first and foremost and create a site that anecdotal evidence suggests does help create community and reduce isolation.

Many factors suggested that the NIATx model might be a useful framework for technology development, such as its basis in years of research about successful change projects, its origin outside the world of user-centered systems design, its simplicity, and its inclusion of a method for arriving at innovative solutions. Although we encountered challenges, we believe the NIATx model is an effective approach to UCD, especially for those not familiar with human factors or UCD principles, and we look forward to trying it again in future projects, as well as continuing to refine the use of the model throughout the development life cycle.

## Abbreviations

AARC: Active Aging Resource Center
ABCD: Asset-Based Community Development
CHESS: The Center for Health Enhancement Systems Studies
ICT: Information and communication technology
NASCAR: National Association for Stock Car Auto Racing
NIATx: Network for the Improvement of Addiction Treatment
PI: Principal Investigator
UCD: user-centered design
